# Horseshoeing1Read before the Pennsylvania State Veterinary Medical Association, March 6, 1900.

**Published:** 1900-04

**Authors:** T. J. Kean

**Affiliations:** Philadelphia, Pa.


### HORSESHOEING.[1]

By T. J. Kean, V.M.D.,
PHILADELPHIA, PA.

I have been requested to read a paper before this Association on "Horseshoeing." It would be impossible in the time limited, as it must be by your patience, to cover the whole ground embraced in the subject of shoeing horses. I have, therefore, prepared a paper on what I consider the cause of all lameness that may be traced directly or indirectly to the shoeing of the horse's foot—that is, the improper distribution of weight on the hoof through the medium of the shoe.

The horse's foot in a state of nature bears the weight of the body transmitted through the leg on the entire bottom or sole of the foot—that is, the wall, bars, sole, and frog each carry their own particular share of weight, acting together as a unit.

When a foot is shod the weight is carried almost wholly on the wall. This is especially true when shod with heel and toe. With a flat shoe over country roads, where there is more or less bedding at each step, we have some weight carried on the frog, but in cities, paved as they now usually are with asphalt or fire brick or on hard telford or macadamized roads, the frogs hardly touch the road surface, to say nothing of bearing weight; accepting the wall and the outer edge of the sole as the part of the foot on which we must depend to support the weight, making use of the frog we can, when we have a frog well enough developed to be of service, we find that the entire circumference of the foot or hoof is intended to carry the same amount of weight normally. While the attachment of the bones to the wall at the heel may not seem as firm as around the front of the foot, it is to be remembered that the heels are reinforced by the curving-in of the bars, by the plantar cushion, and the lateral cartilages, that while the inside wall may be somewhat thinner, weaker, than that of the outside, it at the same time stands more erect, and receives its weight at a better advantage, and though the inner half of the foot has a slightly smaller area

[1] Read before the Pennsylvania State Veterinary Medical Association, March 6, 1900.

than the outside, it is not to be considered as the base of a column only, but in connection with the movement of the limb and the manner in which the weight is transmitted to it. The inside of the foot is straighter along the quarter, but the two heels are to all intents and purposes alike. When standing the weight is evidently distributed, at speed the weight fixes the force of impact with the ground, is received on the heels, and travels progressively forward, leaving the foot of course as it breaks over the toe, so that for all practical purposes all parts of the foot may be considered as carrying the same amount of weight. This is the normal foot, and it is fully capable of standing all the weight that may be thrown on it in the normal, even manner. It is only when the weight becomes disproportionate, when one part is called upon to carry more weight than the others, that we have trouble arising. The most frequent cause of overloading is unilateral contraction and the manner in which such feet are fitted with shoes. Contraction of one heel may be due to heredity or be acquired, and whether it be of the inside or outside heel we have a corresponding reduction in the size of the base on that side—a fewer number of square inches on which to support the normal weight of the limb. This means a greater proportion of pounds to the square inch. If the proportion is too great we have breaking down of the wall at that point and an aggravation of the trouble. The shoes as usually fitted to these feet tend rather to assist in contracting than in correcting them. The horseshoer fits the foot as he finds it, and not as it should be. Instead of studying the limb in connection with the foot, he never sees anything but the hoof.

Say a foot, for illustration, was intended to be three inches across the heel, that it is one and one-half inches from the heel to the centre cleft of the plantar cushion. Say one heel is contracted until it is one-half inch smaller than the other; instead of being one and one-half inches from the centre line to the heel it is only one inch; it is one-third smaller than it should be; one-third smaller than the other side, but it carries the same weight. On hard roads or asphalt this proportion of weight holds good. On a soft road or track, for instance, the ratio of weight carried on the smaller side increases, because of the smaller base of support on that side; there is a tendency for it to sink into the ground; the other side being wider, covering more ground tends to stay more on the surface. This sinking of the one side rotates the foot in that direction; the disproportion between the two sides becomes very much more marked, so much so that quarter-crack is usually produced in this manner. Quarter-crack is by no means the only trouble or even the most probable cause of lameness you will get from a contracted heel and shoe. I speak of it only to show what may happen in extreme cases, though I have never seen a quarter-crack that happened in a race but what was produced in this manner. A contracted shoe—that is, a shoe where one of the heels of which is turned under the foot—will cause an overloading of the heel otherwise normal. You may find an inflammation of any or all the tissues of the overloaded side ; it may manifest itself as a corn on the branch of the sole between the bar and wall, due to bruising the velvety tissue between the bone and the shoe. In these cases we have a state of affairs where the veterinarian and the horseshoer must work together. The veterinarian must supervise the shoeing if he is to meet with success in his treatment. The horseshoer being responsible for the primary condition, can hardly be expected to correct it unaided, nor can the veterinarian hope for permanent results if the horseshoer is permitted to continue the cause. I wish to warn you against the habit some men have of digging or cutting out corns. The veterinarian should impress on the horseshoers he comes in contact with that corns are not the primary cause of lameness; it is simply a symptom, and is to be treated by removing the cause. There can be no good accomplished by digging out a dry corn and burning the velvety tissue with strong acids. If the area is inflamed the whole branch of the sole is to be dressed with the knife, thinned down to allow for swelling of the soft tissue underneath, and prevent pressure and suppuration. If suppuration has already taken place, of course it must be opened and drainage established. The principal in these cases (I am speaking of lameness from overloading without suppuration) is the shoe. How is it fitted and applied ? The shoe in most of the cases will be found to be too short. It may, however, be too long, and may have been turned under the foot because the shoer feared it would be pulled off. The shoe should be of generous length and width of web, the bearing surface at the heels horizontal, and be so fitted to the foot that when examined from behind, the foot on the ground, the heels are an equal distance on each side of a line dividing the bones of the fetlock and pasterns into two equal parts, not an equal distance from the cleft of the frog, as the frog may not be proportionate. If one or other heel is wired or turned in the heel of the shoe must occupy the position that the heel of the foot would have had it been normal. The small heel is to be left off of the shoe one-sixteenth to an eighth of an inch, the foot packed, tubbed, or poulticed. There are other conditions arising from overloading of a part of the foot (but I have intruded on your time long enough): the front of the foot too high; a heel or both heels from too much paring; too short a shoe or too long a toe.

